# Adherence to Combination Prophylaxis for Prevention of Mother-to-Child-Transmission of HIV in Tanzania

**DOI:** 10.1371/journal.pone.0021020

**Published:** 2011-06-10

**Authors:** Inga Kirsten, Julius Sewangi, Andrea Kunz, Festo Dugange, Judith Ziske, Brigitte Jordan-Harder, Gundel Harms, Stefanie Theuring

**Affiliations:** 1 Institute of Tropical Medicine and International Health, Charité-Universitätsmedizin Berlin, Berlin, Germany; 2 Regional AIDS Control Program Mbeya Region, Ministry of Health and Social Welfare, Dar es Salaam, Tanzania; 3 Kyela District Hospital, Ministry of Health and Social Welfare, Dar es Salaam, Tanzania; University of Cape Town, South Africa

## Abstract

**Background:**

Since 2008, Tanzanian guidelines for prevention of mother-to-child-transmission of HIV (PMTCT) recommend combination regimen for mother and infant starting in gestational week 28. Combination prophylaxis is assumed to be more effective and less prone to resistance formation compared to single-drug interventions, but the required continuous collection and intake of drugs might pose a challenge on adherence especially in peripheral resource-limited settings. This study aimed at analyzing adherence to combination prophylaxis under field conditions in a rural health facility in Kyela, Tanzania.

**Methods and Findings:**

A cohort of 122 pregnant women willing to start combination prophylaxis in Kyela District Hospital was enrolled in an observational study. Risk factors for decline of prophylaxis were determined, and adherence levels before, during and after delivery were calculated. In multivariate analysis, identified risk factors for declining pre-delivery prophylaxis included maternal age below 24 years, no income-generating activity, and enrolment before 24.5 gestational weeks, with odds ratios of 5.8 (P = 0.002), 4.4 (P = 0.015) and 7.8 (P = 0.001), respectively. Women who stated to have disclosed their HIV status were significantly more adherent in the pre-delivery period than women who did not (P = 0.004). In the intra- and postpartum period, rather low drug adherence rates during hospitalization indicated unsatisfactory staff performance. Only ten mother-child pairs were at least 80% adherent during all intervention phases; one single mother-child pair met a 95% adherence threshold.

**Conclusions:**

Achieving adherence to combination prophylaxis has shown to be challenging in this rural study setting. Our findings underline the need for additional supervision for PMTCT staff as well as for clients, especially by encouraging them to seek social support through status disclosure. Prophylaxis uptake might be improved by preponing drug intake to an earlier gestational age. Limited structural conditions of a healthcare setting should be taken into serious account when implementing PMTCT combination prophylaxis.

## Introduction

Worldwide, more than two million children younger than 15 years are HIV positive, with 90% of those living in Subsaharan Africa. 370,000 children were newly infected in 2009 [1], mostly by mother-to-child-transmission (MTCT) during pregnancy, during delivery or after delivery via breastfeeding. Without medical intervention, transmission rates range between 25% and 48% in resource-limited settings [2].

For prevention of mother-to-child transmission of HIV (PMTCT), administration of a single dose of the non-nucleoside reverse transcriptase inhibitor (NNRTI) nevirapine (NVP) to both mother and newborn has shown to reduce the transmission risk by over 40% [3], [4]. Single-dosed NVP (sdNVP) is cheap and easy to administer [4]. However, it has been shown that transmission reduction is considerably more effective when combining sdNVP with two nucleoside reverse transcriptase inhibitors (NRTIs), such as zidovudine (AZT) and lamivudine (3TC) [5], [6]. At the same time, sdNVP is prone to resistance formation and might impede subsequent treatment involving NVP or other NNRTIs [7], [8], while combining NVP with NRTIs has shown to reduce the emergence of NNRTI-resistant mutations [8], [9]. Since 2006, the World Health Organization (WHO) therefore recommends a triple combination prophylaxis regimen consisting of two NRTIs (antenatal AZT, intra/postpartum AZT+3TC) and one NNRTI (intrapartum sdNVP) as the standard PMTCT regimen wherever this is feasible [10].

The United Republic of Tanzania is one of the poorest and least developed countries in the world [11], and has an overall HIV prevalence of about 6% [12]. HIV prevalence in pregnant women is estimated at 10–16% [13]. In 2008, the Tanzanian Ministry of Health followed the 2006 WHO guidelines for PMTCT and changed its national standard recommendation from sdNVP to combination prophylaxis. The recommended regimen includes AZT 300 mg twice a day starting in week 28 of pregnancy or as soon as possible thereafter. With the onset of labor, women should take sdNVP, AZT 300 mg every 3 hours, and 3TC 150 mg every 12 hours until delivery. After delivery, a postpartum tail of AZT 300 mg and 3TC 150 mg twice a day should be continued for seven days. All newborns of HIV-positive mothers should receive 2 mg/kg sdNVP within 72 hours and a postpartum tail of 4 mg/kg AZT twice a day for seven days if the mother took AZT during pregnancy for four weeks or longer. Otherwise, the infant postpartum tail should last for four weeks. In both the Tanzanian and WHO recommendations, sdNVP only remains the minimum prophylactic standard for PMTCT if more complex interventions are not feasible [14]. Notably, while Tanzanian guidelines at time of study conduction were based on 2006 WHO recommendations, those were again revised in 2010. WHO now recommends start of AZT intake from gestational week 14 onwards, suggesting that an omission of sdNVP can be considered if AZT was taken for more than four weeks before delivery [15].

Optimal drug adherence is crucial for drug effectiveness: on the one hand, to sufficiently suppress the maternal viral load [16], which in turn is one of the most important risk factors for MTCT [17]. On the other hand, maladherence to antiretroviral drugs potentially promotes the emergence of resistant viral strains which may lead to failure of subsequent treatment [18].

Studies regarding adherence to combination prophylaxis for PMTCT in rural, structurally limited settings are largely lacking up to today. However, previous research on the acceptance of simpler PMTCT regimens showed that in peripheral settings of Tanzania, Uganda and Zambia, sdNVP was accepted by only 20–60% of HIV-positive women seeking antenatal care (ANC) [4], [19]. This allows for hypothesizing that more complex regimens might inherit an even greater risk of suboptimal adherence in rural areas, and that combination PMTCT prophylaxis might not bring the expected benefit compared to sdNVP in terms of reduced transmission rates and emerging resistance. The aim of this study has been to gain insight into adherence to combination prophylaxis in one exemplary district hospital in rural Tanzania, and to identify possible challenges of adherence when implementing this regimen.

## Methods

The study was undertaken at Kyela District Hospital (KDH), which is a rural health facility in Mbeya Region, Tanzania, and has been providing PMTCT services supported by the German Agency for Technical Co-operation since 2001. There are approximately 130 pregnant women who approach KDH as new ANC clients every month. While adult HIV prevalence in Mbeya Region is found at around 8% [20], HIV prevalence reached 18% among pregnant women in KDH in 2007 (unpublished data). Combination prophylaxis was introduced as the standard PMTCT regimen in KDH in March 2008. KDH was the only facility offering the extended regimen in the vicinity at time of study conduction, while other closeby facilities offered sdNVP only. Our study observed the routine ANC-embedded PMTCT procedure in KDH between October 2008 and September 2009.

The procedure comprised provider-initiated voluntary HIV-counseling and testing of all pregnant women approaching ANC services. Couple testing was encouraged. In HIV-positive women, CD4-cell count was performed to assess indication for antiretroviral therapy (ART). Those with a CD4-cell count<200 cells/µl at stage 1 or 2 of the WHO clinical staging [21], CD4-count<350 cells/µl at stage 3 and women at stage 4 regardless of CD4-count were referred to ART. These women were automatically ineligible for study participation. Women not indicated for ART were offered combination prophylaxis according to the new Tanzanian guidelines if not showing contraindications like severe anemia (hemoglobin<7.5 g/dl) or granulocytopenia (granulocytes<0.75*10^9^/l) [14]. Consenting to take part in the prophylaxis intervention was considered as PMTCT enrollment. The PMTCT intervention required women to start AZT intake in gestational week 28, or as soon as possible thereafter. According to Tanzanian PMTCT guidelines, women were scheduled to collect their AZT supply weekly at KDH for the first month of prophylaxis and monthly thereafter. Drug collection required an ANC visit and a subsequent visit of KDH pharmacy.

All women were strongly advised to deliver in KDH, or to return to KDH within 72 hours postpartum if delivery elsewhere was unavoidable. To ensure sdNVP intake at the onset of labor regardless of the place of delivery, women having reached gestational week 28 were handed out maternal sdNVP for possible self-administration. For deliveries in KDH, maternity staff dispensed drugs to mothers and newborns during hospitalization. At discharge, mothers received AZT and 3TC tablets and infant AZT syrup for the remaining postpartum tail to take home. In the case of delivery elsewhere, postnatal infant sdNVP and maternal and infant postpartum drugs to take home were dispensed upon women's return within 72 hours. Women who had not started prophylaxis during pregnancy, but came to KDH for or after delivery, were equally offered intra/postpartum prophylaxis.

Between October 2008 and July 2009, PMTCT-enrolling women were systematically included in the observational study if they fulfilled the eligibility criteria of written informed consent, >18 years of age and no signs of psychological disorder. Confidentiality of all obtained data was ensured. Study participation did not involve additional hospital visits beyond those necessary for the PMTCT intervention.

### Data collection

Standardized questionnaires for the stages of ANC, delivery and maternity were developed by the authors and modified during a pretesting period of one week before start of the study.

Data collection included an initial assessment of sociodemographic, socioeconomic and clinical parameters at the point of consenting to participate in the study. Observations were then divided into a pre-delivery and an intra/postpartum period, giving consideration to respective different implications for adherence, i.e. client-driven drug collection during pregnancy and provider-driven intra/postpartum drug dispensation. All stages of data collection were supported by a trained study nurse, ensuring the routine workflow would not be disturbed.

For pre-delivery data collection, study participants were interviewed during their recurrent ANC visits until delivery. The study nurse conducted interviews in Swahili or the local language, and took down answers in English. Pre-delivery questionnaires focused on aspects like drug adherence, status of HIV-disclosure, and reasons for failed drug collection in case of one or more missed drug collection episodes. General acceptance of prophylaxis among study participants was defined as having collected AZT at least once in the pre-delivery period. Those who never collected AZT during pregnancy were considered to be declining prophylaxis. The definition of acceptance and decline was limited to the pre-delivery period because it was assumed that drug receipt during intra/postpartum hospitalization would not to the same extent reflect an active acceptance process.

Intra/postpartum data collection included all study participants who returned to KDH for delivery or within 72 hours thereafter. Those who had declined pre-delivery prophylaxis, but returned to KDH intra/postpartumly were equally incorporated into this part of observation. Maternity ward nurses, supported by the study nurse, were trained to fill in questionnaires listing all different drugs dispensed in the intra/postpartum period for each woman and newborn, including postpartum tail drugs handed out to take home. Intrapartum AZT and 3TC administration was only observed in KDH deliveries. Study participants who had delivered outside of KDH reported self-administration of sdNVP to staff at their postpartum return; their questionnaires were then continued with information on infant sdNVP intake and dispensed take-home drugs.

Study participants who did not return to KDH for delivery or within 72 hours postpartum were defined as lost to follow-up.

### Definition and assessment of adherence

As customary for PMTCT services, drug intake was not directly observed in most parts of the intervention. Accordingly, adherence measures assessed drug collection and dispensation rather than actual ingestion, except during intra/postpartum hopitalisation. Overall adherence was defined to consist of women's drug collection during pregnancy (pre-delivery adherence), and drug dispensation by staff during/after delivery including the postpartum take-home tail (intra/postpartum adherence).

Pre-delivery adherence was measured by women's medication possession ratio (MPR) [22], which was generated from the number of collected AZT doses divided by the targeted number of doses between start of prophylaxis and delivery. Thus, collecting all drugs as scheduled yielded an MPR of 100%, defined as full adherence until delivery. In case of an unknown date of delivery, an estimated delivery date was used to calculate the MPR.

Assessing intra/postpartum adherence was based on staff-listed dispensation of respective drugs. Adherence to sdNVP was measured in absolute intake numbers for mothers and newborns as reported by nurses, or reported by women in case of maternal self-administration. For maternal intrapartum AZT and 3TC, adherence rates were assessed by dividing the hours covered through dispensed drugs by the total hours of hospitalization until delivery. Postpartum tail adherence comprised two parts: first, the observed dispensation of AZT and 3TC to mothers and AZT to newborns before hospital discharge, measured by hours of hospitalization covered with drugs, and second, dispensation of take-home doses for mother and infant after hospital discharge, measured in absolute numbers of women and infants supplied with the correct amount of drugs to take home. Outcome measures of the different observed stages are summarized in Table 1.

**Table 1:** Summary of used adherence outcome measures.

Observation phase	Subgroup	Drugs	Adherence outcome measure
**Antenatal**	Maternal	AZT	Medication possession ratio from women having started prophylaxis in ANC
**Intrapartum**	Maternal	sdNVP	Intake ratio from all women observed intra/postpartumly
	Maternal	AZT/3TC	% of hours covered with drugs from total hospitalization hours between admission and delivery*
**Postpartum**	Maternal	AZT/3TC	• % of hours covered with drugs from total hospitalization hours between delivery and hospital discharge*
			• % of women having received their correct take-home dose from all women observed intra/postpartumly
	Infant	sdNVP	Intake ratio from all surviving newborns observed postpartumly
	Infant	AZT	• % of hours covered with drugs from total hospitalization hours between delivery and hospital discharge*
			• % of infants who received their correct take-home dose from all surviving infants observed postpartumly

*among hospital deliveries.

### Statistical methods

Data analysis was performed with a standard statistical software package. For dichotomization of metric parameters like age and gestational age at enrollment, cut-off-thresholds were defined by using JRip, which is part of the open source machine learning software WEKA 3 [23] and identifies thresholds with the most discriminative power regarding the dependent variable. We set a CD4-count cut-off level at ≤350 cells/µl due to the relevance of this value in international guidelines [15]. For the remaining interval variables, the median was used as cut-off threshold. Pearson's chi square test and Fisher's exact test were used to compare categorical data. To evaluate socio-demographic variables and their influence on declining adherence, univariate odds ratios were calculated. Variables that showed to be significant in the univariate analysis (P<0.05) were included into a multivariate analysis using a forward stepwise logistic regression to calculate adjusted odds ratios. For analysis of adherence levels, adherence cut-off points of ≥95% and ≥80% were used for comparability with other studies [24]. The influence of sociodemographic variables on adherence values was tested using the Mann-Whitney U-test.

### Ethical considerations

The study was conducted in accordance with the Declaration of Helsinki. It was approved by the Tanzanian National Institute for Medical Research, by the Ethical Committee Mbeya, and by the Ethical Committee of Charité-Universitätsmedizin Berlin, Germany. Written informed consent in Swahili was obtained from all participants. Confidentiality was maintained throughout the study.

## Results

In total, 1395 women were counseled and HIV-tested in ANC of KDH during the study period. Among 202 women identified HIV-positive (14.5% of all tested), 72 (35.6%) did not meet the eligibility criteria of the study, mostly because of an ART indication. From 130 eligible women, 5 moved away and were therefore referred to another health facility, 2 had an abortion before starting prophylaxis, and 1 withdrew her informed consent.

For the remaining cohort of 122 study participants, the following median demographics were determined: 26 years of age (range 18–37 years), 23 weeks of gestational age at PMTCT enrollment (range 9–36 weeks), 7 years of education duration (range 0–12 years), 30 minutes travel time to KDH (range 0–120 minutes), and 0 Tanzanian Shilling of travel costs to KDH (range 0–2000 Tanzanian Shilling). Seven women (5.7%) were counseled and tested together with their partner. Women's median CD4-cell count reached 391 cells/µl (range 200–883 cells/µl).

**Figure 1:**
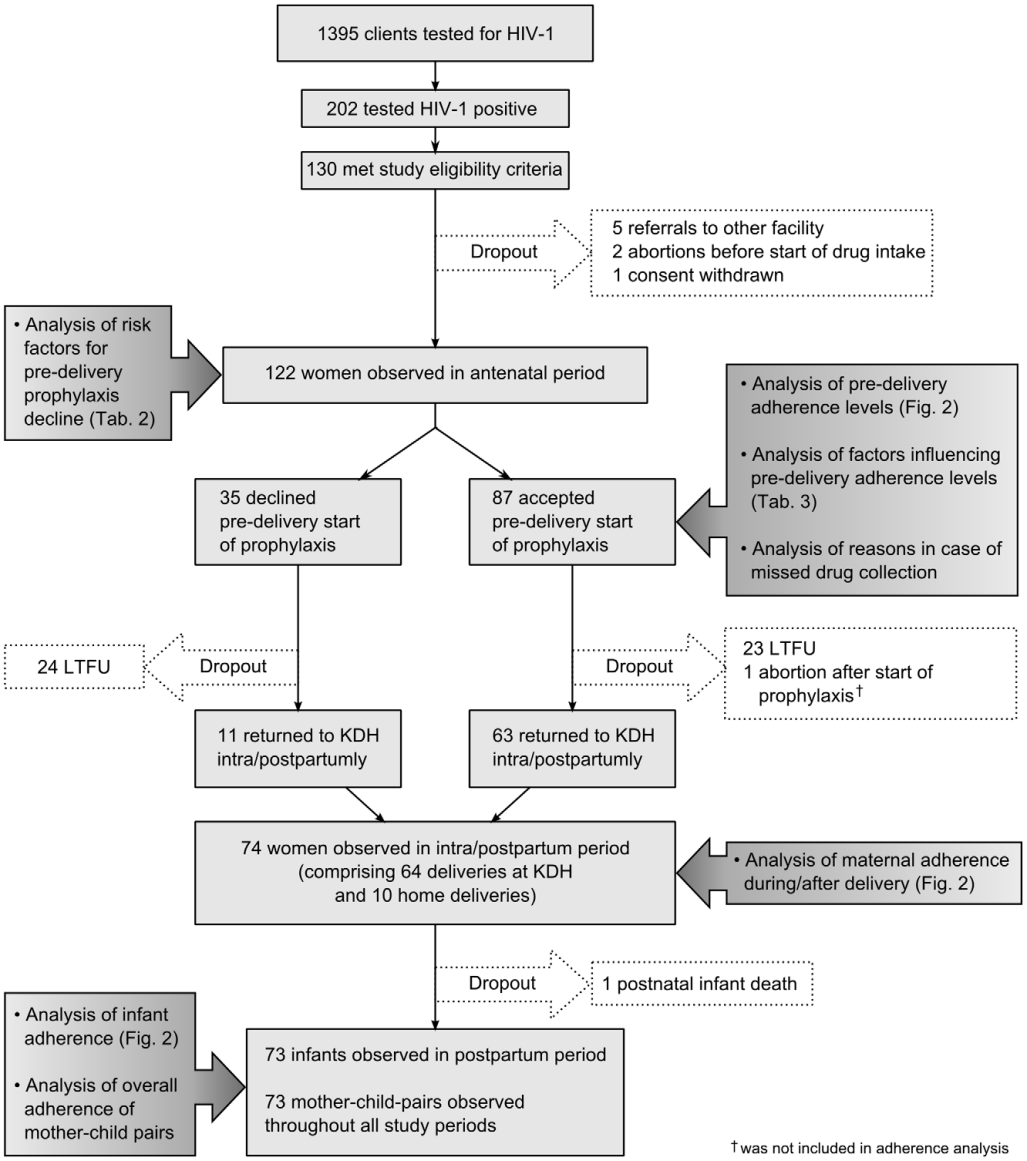
Profile of cohort and analyses. This figure is illustrating the formation of the cohort and of its different subgroups, including women observed in the antenatal period, women accepting/declining antenatal prophylaxis, women lost to follow-up (LTFU), women observed intra/postpartumly and infants observed postpartumly. It is indicated which analyses have been performed within the respective subgroups and where the results of those analyses are shown.

Forty-seven of 122 participants (38.5%) were lost to follow-up. Among these were 24 women who declined prophylaxis from the beginning, while 23 started prophylaxis, but failed to return to KDH. One woman had an abortion after starting prophylaxis and one baby died shortly after delivery. Seventy-three mother-child pairs were observed from the point of maternal PMTCT enrollment to the observation endpoint of maternal and infant drug supply in the intra/postpartum period. Figure 1 shows an overview of all undertaken analyses in different subgroups and respective drop-outs.

### Factors associated with declining combination prophylaxis

Out of the 122 enrolled women, 35 (28.7%) declined pre-delivery prophylaxis. From these decliners, 11 returned to KDH in the intra/postpartum period.

In bivariate analysis, variables associated with declining pre-delivery prophylaxis were women's age of 23 years or below (P<0.001), no income-generating activity (P = 0.006), and a gestational age at enrollment of 24.5 weeks or below (P<0.001). In multivariate analysis, these variables showed to be associated independently with the decline of prophylaxis with P values of 0.002, 0.015, and 0.001, respectively. As shown in Table 2, other sociodemographic, economic and health-related variables, like education, travel distance or CD4 cell count, were not significantly associated with decline.

**Table 2:** Risk factors for decline of pre-delivery prophylaxis.

			Bivariate					Mulitvariate	
Variable	n	decline	OR	0.95 CI		P Chi^2^		AOR	P
**Age** ‡	**122**								
>23 years	91	17.58%	1.000					1.000	
≤23 years	31	61.29%	7.422	3.011–	18.292	<0,001	*	5.841	0.002
**Education**	**113**								
<7 years	19	21.05%	1.000						
≥7 years	94	28.72%	1.511	0.460–	4.967	0.494			
**Income-gen. activity**	**116**								
Yes	44	13.64%	1.000					1.000	
No	72	37.50%	3.800	1.420–	10.169	0.006	*	4.399	0.015
**Marital status**	**114**								
Single	24	25.00%	1.000						
Married	90	28.89%	1.182	0.422–	3.308	0.750			
**Gravida**	**122**								
Multigravida	113	28.32%	1.000						
Primigravida	9	33.33%	1.266	0.298–	5.369	0.719	**		
**Gestational age** ‡	**122**								
>24.42 weeks	52	9.62%	1.000					1.000	
≤24.42 weeks	70	42.86%	7.050	2.501–	19.874	<0,001	*	7.820	0.001
**Last delivery**	**98**								
At home	27	29.63%	1.000						
At health facility	71	32.39%	1.138	0.434–	2.984	0.793			
**Travel distance**	**121**								
≤30 minutes	64	26.56%	1.000						
>30 minutes	55	32.73%	1.345	0.610–	2.965	0.462			
**Transport costs**	**117**								
No	74	28.38%	1.000						
Yes	43	30.23%	1.094	0.480–	2.493	0.831			
**CD4 cell count**	**122**								
>350	50	26.00%	1.000						
≤350	72	30.56%	1.252	0.559–	2.806	0.584			

OD = odds ratio; CI = confidence interval; AOR = adjusted odds ratio.

‡Threshold calculated with software JRip.

*Variables included into multivariate analyses.

**Fisher's exact test.

### AZT-adherence levels before delivery

**Figure 2:**
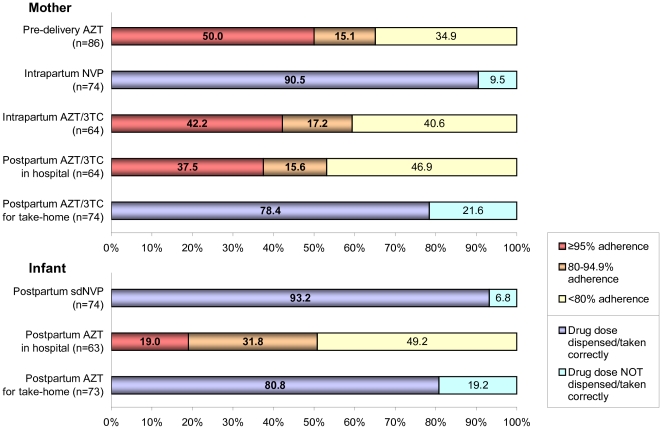
Adherence levels reached for different drugs in antenatal, intrapartum and postpartum period. Adherence cut-off levels of 95% and 80% are described for women's adherence to antenatal AZT, intrapartum AZT+3TC and postpartum AZT+3TC during hospitalization, as well as for infant adherence to AZT during hospitalization. For pre-delivery AZT intake, an adherence level of ≥95% was reached by 50.0% and a level of ≥80% was reached by 65.1% of women. For intrapartum AZT+3TC, adherence of ≥95% was reached by 42.2% of women who delivered in KDH and adherence of ≥80% was reached by 59.4% of them. For postpartum AZT+3TC during hospitalization, adherence of ≥95% was reached by 37.5% and adherence of ≥80% was reached by 53.1% of hospital-delivering women. In newborns, AZT adherence during hospitalization was ≥95% in 19.0% and ≥80% in 50.8% of the infants. For sdNVP, proportions reflect self-reported correct intake of drug doses for mothers (90.5%) and staff-reported correctly dispensed drug doses for newborns (93.2%). Postpartum take-home doses were dispensed correctly to 78.4% of women and 80.8% of infants.

From 87 observed women having accepted prophylaxis, one subsequently had an abortion and was excluded from analysis (Figure 1). Among the remaining 86, the mean AZT-adherence before delivery was 77%. 56 women (65.1%) attained at least 80% adherence, and 43 women (50.0%) reached at least 95% adherence (Figure 2). Full adherence until delivery was achieved by 33 women (38.4%) who collected all AZT doses as required.

Disclosure of the HIV-status to the partner, a relative or a friend was positively associated with adherence rates before delivery: out of the 86 women who had started to take prophylaxis, 6 (7.0%) had not disclosed their HIV status and were significantly less (median 22.7%) adherent than women who had disclosed their status (median 97.3%, P = 0.004). Other variables were not significantly correlated with adherence levels (Table 3).

**Table 3:** Influence of sociodemographic, economic and health-related factors on pre-delivery AZT adherence level.

Variable		Antenatal adherence	
	n	median	P*
**Age**	**86**	**93.92%**	0.558
≤23 years	11	95.08%	
>23 years	75	90.74%	
**Education**	**81**	**96.84%**	0.320
<7 years	15	87.16%	
≥7 years	66	97.86%	
**Income-generating activity**	**82**	**93.92%**	0.856
Yes	38	90.17%	
No	44	97.26%	
**Marital status**	**84**	**95.40%**	0.848
Single	18	93.92%	
Married	66	96.28%	
**Gravida**	**86**	**93.92%**	0.834
Primigravida	6	95.40%	
Multigravida	80	90.68%	
**Gestational age**	**86**	**93.92%**	0.176
≤24.42 weeks	47	87.76%	
>24.42 weeks	39	98.11%	
**Last delivery**	**66**	**95.96%**	0.278
At home	19	87.16%	
At health facility	47	96.84%	
**Travel distance**	**83**	**95.08%**	0.788
≤30 minutes	46	90.68%	
>30 minutes	37	97.78%	
**Transport costs**	**83**	**95.71%**	0.728
Yes	30	97.86%	
No	53	90.63%	
**CD4 cell count**	**86**	**93.92%**	0.304
≤350	36	98.48%	
>350	50	89.59%	
**Disclosure**	**86**	**93.92%**	0.004
Stated	80	97.26%	
Not stated	6	22.69%	

*Mann-Whitney U-Test.

Overall, 53 out of 86 participants (61.6%) were not fully adherent, i.e. had missed at least one drug collection episode. In 34 of these (64.2%), reasons for failure in drug collection were unknown, partly because they did not state any reason when asked (11 women), and partly because they could not be asked due to loss to follow-up (23 women). Among the 19 women who had stated one ore more reasons (24 reasons mentioned altogether) for occurred missed drug collection episodes, personal reasons were mentioned most frequently (14/19 women, 73.7%), and included family obligations (mentioned by 6 women), forgetting appointments (mentioned by 4), transportation difficulties (mentioned by 3), and incorrect dosing of tablets (one mention). At the same time, 10 of the 19 women (52.6%) named hospital-based reasons, i.e. incorrectly indicated dates for the next visit (five mentions), neglected dispensation of drugs at KDH pharmacy (three mentions), or staff had decided to interrupt prophylaxis (two mentions).

### Adherence during and after delivery

Out of 122 study participants, 74 were being observed in the intra/postpartum period (Figure 1). From these, 63 (85.1%) had accepted pre-delivery prophylaxis, while 11 (14.9%) women had declined. Pre-delivery prophylaxis acceptance was significantly associated with returning to KDH during or within 72 hours after delivery (P<0.001).

Sixty-four of 74 intra/postpartum observed participants (86.5%) delivered in KDH, and 10 women (13.5%) delivered at home but returned to KDH within 72 hours postpartum.

SdNVP was taken by 67 out of the 74 women (90.5%) in the correct time frame before delivery, 59 of these delivered in KDH, and eight delivered at home. Sixty-nine newborns (93.2%) received postnatal sdNVP in KDH, including 60 (93.8%) out of 64 KDH deliveries and 9 (90.0%) out of 10 home deliveries.

Regarding intrapartum AZT and 3TC, of the 64 deliveries in KDH, 38 women (59.4%) attained at least 80% and 27 (42.2%) at least 95% adherence. 26 women (40.6%) remained below 80% adherence. Between the time of delivery and hospital discharge, 34 of the 64 women (53.1%) reached 80% and 24 (37.5%) reached 95% adherence. From the 63 surviving newborns, for 32 (50.8%) at least 80% adherence was achieved, and for 12 children (19.0%) at least 95% adherence was achieved when starting the postpartum AZT tail during hospitalization.

From the 74 women which had either delivered in KDH or returned there within 72 hours, 58 (78.4%) received the correct amount of AZT and 3TC-tablets to take home, and 59 infants (80.8% from 73 surviving) were dispensed the correct take-home amount of AZT syrup. Achieved intra/postpartum adherence levels for mothers and infants are depicted in Figure 2.

### Overall adherence before, during and after delivery

Initially, 122 women had agreed to take combination prophylaxis. One had an abortion after starting prophylaxis and one child died shortly after delivery. Out of the remaining 120 mother-child pairs, 10 (8.3%) achieved at least 80% adherence rates in all phases before, during and after delivery, and only one single mother-child pair (0.8%) achieved a 95% adherence level for the entire PMTCT intervention.

## Discussion

Our observational study in a rural setting in Tanzania found relatively low levels of adherence to combination prophylaxis in all different PMTCT intervention phases.

Within the study population, young age and no income-generating activity of the women were found to increase the risk of not starting combination prophylaxis before delivery. This is in accordance with other studies, which identified similar risk factors to influence the acceptance of ART [25] and of sdNVP-based interventions [19]. Gestational age below 24 weeks at PMTCT enrollment was discovered as an additional risk factor for declining combination prophylaxis. An explanation for this could be the time gap emerging for women who become enrolled in an early pregnancy stage, but then have to wait until gestational week 28 until they can actually start prophylaxis. This time gap before drug intake might imply that antiretroviral prophylaxis has no acute priority, and forgetting future appointments may be likely. In response to this problem, revised WHO-guidelines of July 2010 recommend prophylaxis start from gestational week 14 onwards, aspiring to avoid idle time between enrollment and first drug intake [15]. Although this requires adhering to antenatal AZT for an even longer period of time, facilitating immediate prophylaxis start for the case of early PMTCT enrollment will simplify the PMTCT procedure to some extent and should be transferred into national guidelines as soon as possible. Yet, it will be of high importance to keep track of possibly increased resistance formation arising from extended antenatal AZT monotherapy.

Among those who started combination prophylaxis, every third woman remained below an 80% adherence level in the period before delivery. This rate underachieves results of a similar study on ART adherence in a Zambian cohort of patients receiving treatment for their own health, where only about 15% of patients remained below an 80% adherence level [24]. In a previous meta-analysis on ART adherence among general population receiving treatment, it was reported that in 20 out of 22 studies, more than half of the patients met the thresholds of 95% or full adherence [26]. While those ART-related studies suggest that higher adherence levels are achievable also in resource-limited settings, general ART programs can only represent an approximate comparative value with regards to PMTCT, because the subgroup of pregnant women involves specific implications for adherence. More research, filling the existing knowledge gap on adherence to PMTCT combination prophylaxis, is desirable to allow for putting our findings into context.

In our study, the only factor significantly influencing levels of pre-delivery adherence was HIV-status disclosure to the partner, a relative or a friend. Status disclosure is known to relieve women from emotional stress and to enable them to receive psychological and material support by their social environment [27]. Especially in rural settings, disclosure can facilitate issues like obtaining approval from the husband as the main decision maker to seek healthcare, and transportation to the hospital. From women who started to take prophylaxis, 93% disclosed their status, a high rate compared to other studies [28], [29]. However, disclosure information could only be obtained from women who returned to ANC at least once after having been HIV-tested. Given that prophylaxis start is positively influenced by disclosure [29], our rate is likely to be overestimated due to the exclusion of 35 women declining prophylaxis.

Couple counseling and HIV-testing often facilitates partner disclosure and positively influences general uptake of PMTCT programs [30], [31]. Deplorably few of our study participants were counseled and tested together with their partner, confirming the prevalent observation that adequate male involvement in PMTCT is still largely missing [32]. A stronger implementation of couple counseling and testing, and encouraging pregnant women to seek support in their social environment through status disclosure, not only to partners, but also to other confidants, e.g. in the form of treatment buddies or community groups, could be effective strategies for achieving better adherence rates [33], [34].

Failure in pre-delivery adherence stands for women's missed or delayed hospital visits to collect drugs, which has been described as one of several causes for maladherence in other studies based on self-reported adherence rates to ART among general population receiving treatment for their own health [35], [36]. We do not know the reasons for missed drug collection episodes in the majority of our study participants, either because they did not return to be asked, or because they did not want to state reasons. In those who shared their reasons for failure in collecting drugs, personal obstacles were mentioned most frequently, but every second of those women also stated that she failed to get drugs because of a wrongly scheduled date for the next drug collection or reluctance of drug dispensation by KDH staff. This demonstrates the crucial importance of high quality training of hospital staff [37].

Staff were also responsible for providing correct doses of AZT and 3TC at the right time during hospitalization of mother and newborn. For only about half of the mothers and babies, at least 80% adherence was achieved. The threshold of 95% adherence was met for less than half of the mothers and only one fifth of the babies. At hospital discharge, only about 80% of mothers and babies received the right amount of take-home drugs. These intra/postpartum adherence levels indicate suboptimal staff performance, and might partly be explained by the increased burden of work due to the introduction of combination prophylaxis. High workload can decrease motivation and dedication of staff, and might result in inadequate care. Systems of rotating staff members between departments often cause additional shortage and require continuous training of new PMTCT staff [37], [38]. Reducing staff rotation and ensuring a close professional supervision could mitigate the shortage of well-trained personnel, and thereby considerably contribute to improved adherence rates during hospitalization. At the same time, it should be noted that in some other African countries, e.g. Kenya or Malawi, national guidelines recommend a simplified intrapartum regimen, administrating one dose of 600 mg AZT or 300 mg AZT twice daily instead of the three-hourly dosing throughout labor [39], [40], a practice which could also reduce intrapartum care-related staff burden to some extent.

More than one third of the enrolled women were lost to follow-up, which is a larger fraction than in comparable studies on ART and sdNVP adherence [41], [42]. Notably, only 10 mother-child pairs achieved at least 80% adherence in all phases of combination prophylaxis, and only one pair was adherent on a 95% level. We have targeted adherence levels of 80% and 95% for best comparability with other findings, but in fact there is a lack of evidence on necessary adherence levels to ensure the effectiveness of combination prophylaxis and to prevent emergence of resistant viral strains. In PMTCT regimen trials like the Kesho Bora study, detailed adherence levels among the study population have generally not been determined, while the trials have rather considered percentages of women who reached full adherence [43], or, like in the DITRAME trial, it was only assessed how many women met a certain threshold like 80% adherence [44]. Further research on specific necessary thresholds would be highly desirable for a more revealing interpretation concerning implications of adherence levels on drug effectiveness and resistance formation.

In the near future, the Tanzanian government will very likely align national PMTCT guidelines with WHO's 2010 revised recommendations, including the extension of ART-eligibility to those women with a CD4 count below 350 instead of 200, and including ARV intake throughout the entire breastfeeding period for infant (option A) or mother (option B) [15]. This will imply new challenges with regards to adherence, particularly for women choosing to breastfeed. According to WHO, 96.4% of Tanzanian infants are ever breastfed, with median breastfeeding duration of 21.1 months [45]. This is in compliance with PMTCT monitoring data from Mbeya Region, where over 95% of HIV positive women preferred breastfeeding to formula feeding (2009, data unpublished). Hence, for a mainly breastfeeding population like in our study setting, the chosen regimen option recommended in subsequent Tanzanian guidelines will be a highly relevant issue in the upcoming years.

An important limitation of the study is that pre-delivery adherence values in our analysis are based on MPRs. Although found to be a valid measurement for the reduction of viral load [46], [47], MPRs bear the risk of overestimating real adherence values [48], [49], because the amount of handed-out drugs might differ from the amount of actually ingested drugs. Suggesting that fewer women than assumed might actually have taken drugs as required, this would, however, strengthen our finding of suboptimal adherence.

Although we have chosen a representative district hospital strictly oriented in national guidelines, local customs might cause a systematical bias, and further research is strongly needed to assess the validity of our findings for other regions.

In conclusion, achieving high adherence rates during all phases of PMTCT combination prophylaxis has shown to be challenging in the rural setting of this study. Prophylaxis uptake might be improved by preponing drug intake to an earlier gestational age. Our findings underline the need for additional training and supervision for overburdened PMTCT staff as well as close supervision for PMTCT clients, especially by encouraging them to seek social support through status disclosure.

Combination prophylaxis for PMTCT is gradually replacing less effective regimens in many resource-limited regions of the world. However, the finding that only one mother-child pair managed to receive 95% of the intended quantity of drugs in all PMTCT phases in our study site results in a serious derogation of the assumed high effectiveness of combination prophylaxis in rural settings. It should be considered as a liability of health authorities to take limited structural conditions into account when planning, implementing and expanding combination prophylaxis for PMTCT.
